#  10-Year-Old Female with Acute Abdominal Pain with Pancreatic Mass

**DOI:** 10.1155/2017/3253787

**Published:** 2017-10-09

**Authors:** Charles K. Powers, Molly Posa, Dhanashree Rajderkar, Jaclyn Otero

**Affiliations:** ^1^University of Florida COM, 1600 SW Archer Road, P.O. Box 10296, Gainesville, FL 32608, USA; ^2^University of Florida COM, 133 SW 130th Way, Suite C, Newberry, FL 32669, USA; ^3^University of Florida COM, 4740 NW 39th Place, Suite B, Gainesville, FL 32606, USA

## Abstract

A previously healthy 10-year-old female presented to a local emergency department following three days of nausea and vomiting diagnosed with a solid pseudopapillary tumor. Solid pseudopapillary neoplasms are a rare form of pancreatic cystic neoplasm that typically presents in young females in their 20–30s and are very rare in children. These neoplasms often present as an asymptomatic tumor found on incidental imaging. When symptomatic they most commonly present with abdominal pain and can also cause a palpable abdominal mass, weight loss, gastrointestinal obstruction, and nausea and vomiting. Timely diagnosis of this rare neoplasm is very important because complete resection of the tumor is the definitive treatment and leads to an excellent long-term survival.

## 1. Case Presentation

A previously healthy 10-year-old female with no significant past medical history presented to a local emergency department following 3 days of nausea and vomiting with mild diffuse abdominal pain. Her mother reports that the patient had nausea over a 3-day period with frequent episodes of nonbloody, nonbilious emesis. The first day of illness she was vomiting every 20 minutes. The following 2 days she vomited only after eating and drinking. Due to her persistent emesis, her oral intake had significantly decreased by time of presentation. Although normally energetic, she developed significant fatigue. There was no change in mental status. The mother initially believed that her symptoms were infectious in nature as several of her peers also had nausea and vomiting. She was subsequently brought to the emergency department on day 3 of illness due to her fatigue, worsening diffuse abdominal pain, and parental concern for dehydration.

On physical examination, she was afebrile with tachycardia to 120 and mild hypertension of 131/84. On abdominal exam she demonstrated periumbilical and right sided tenderness to palpation. She had normoactive bowel sounds without guarding or rebound tenderness. Psoas and obturator signs were negative. No masses were palpated and she did not have hepatosplenomegaly. The remainder of her physical exam was unremarkable.

Mother denied family history of pancreatic cancer. Maternal aunt had pancreatitis requiring surgical intervention; further details are unknown.

### 1.1. Hospital Course

**Figure 1:**
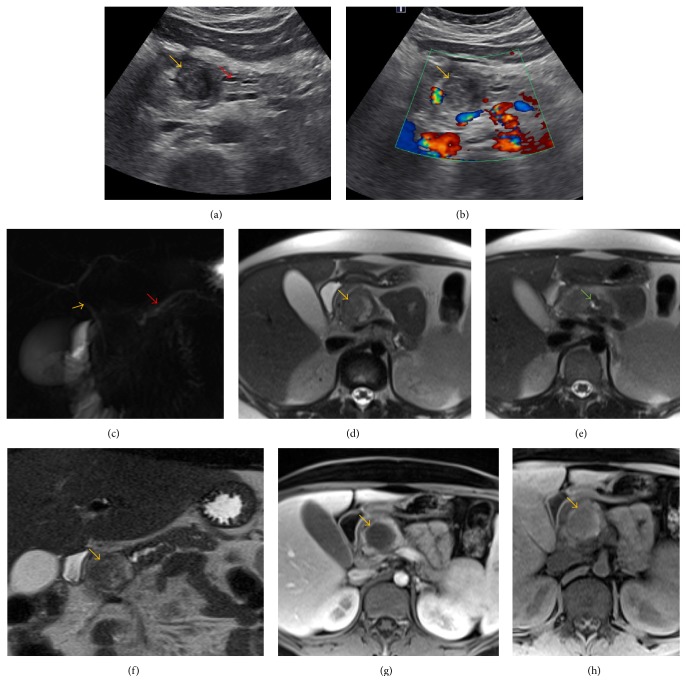
(a and b) Ultrasound images. (a) Well-defined hypoechoeic lesion without necrosis or calcification (orange arrow). Mildly dilated pancreatic duct (red arrow). (b) Color Doppler evaluation showing no color flow within the lesion in the pancreas (orange arrow). (c, d, e, f, g, and h) MRI and MRCP images. (c) MRCP-Normal CBD (orange arrow). Mildly dilated pancreatic duct (red arrow). (d) T2 weighted axial image showing heterogeneous mass in the head of the pancreas (orange arrow). (e) Mildly dilated pancreatic duct (green arrow). (f) Coronal T2 weighted image showing heterogeneous mass in the head of the pancreas (orange arrow). (g) Axial T1 weighted image without contrast showing well-defined hypointense lesion in the pancreas (orange arrow). (h) Axial postcontrast image showing no enhancement of the lesion in the head of the pancreas (orange arrow).

A complete blood count, comprehensive metabolic panel, and lipase were obtained, all of which were normal other than a significantly elevated lipase of 393. An abdominal ultrasound was obtained which showed a well-defined round heterogeneous mass in the head of the pancreas (Figure 1(a)) without significant vascular flow on the Doppler image (Figure 1(b)). The patient was transferred to a pediatric tertiary care center.

Mother denied family history of pancreatic cancer. Maternal aunt had pancreatitis requiring surgical intervention; further details are unknown.

The patient underwent a magnetic resonance cholangiopancreatography (MRCP) the following day for further characterization of the lesion and to evaluate the biliary system. MRI demonstrated a 2.6 × 2.6 cm pancreatic head mass (Figure 1(d)) with heterogeneous internal signal (Figures 1(f) and 1(g)) and main pancreatic duct dilatation to 3.7 mm (Figure 1(e)). There was no significant postcontrast enhancement (Figure 1(h)). The MRCP images demonstrated normal CBD and mildly dilated pancreatic duct (Figure 1(c)). which heightened suspicion for a solid pseudopapillary epithelial neoplasm or cystic endocrine tumor of the pancreas. There was no evidence of the lymph nodal or hepatic metastases.

Four days later an endoscopic ultrasound with fine needle biopsy was performed. Pathology results were consistent with solid pseudopapillary tumor. The patient was discharged until a planned readmission 4 days later at which time she underwent a laparoscopic radical Whipple's (pancreaticoduodenectomy) for removal of the pancreatic mass. The surgery was completed successfully without complication.

## 2. Diagnosis

Pathology of the removed specimen demonstrated solid pseudopapillary tumor of the pancreas with extensive necrosis and negative margins. She recovered well from the procedure and was discharged 6 days later. At her follow-up appointment a month later, she had completely recovered without any sequelae. The mother reports that “it is like nothing ever happened.”

## 3. Discussion

Solid pseudopapillary neoplasms (SPNs) are categorized as a pancreatic cystic neoplasms and SPNs are the least common neoplasm within this group [1]. Cystic pancreatic lesions in general are less rare; an estimated 7% of patients who undergo imaging with MRI or CT will have a pancreatic cyst; however, less than 5% of these will have SPNs [1]. SPNs are rare tumors which are typically seen in females in their 20s and 30s [2]. The male to female ratio is 1 : 10 and the average age of presentation is 22 years old [2]. SPNs are even more uncommon in pediatrics, with an incidence rate of 0.005–0.01 cases per 100,000. Pediatric SPNs have distinct clinical presentation and features summarized in Table 1 [3].

While more symptomatic initial presentations were seen in the past, incidental diagnosis has become the most frequent presentation due to the widespread availability of sensitive cross-sectional imaging [4]. When symptomatic, the most common presenting symptom is abdominal pain. Tumor size ranges from 2 to 20 cm in diameter and 25% of pediatric cases will have a palpable mass at the time of presentation [1, 3]. The third most common presenting symptom, which was seen in our patient, is nausea and vomiting. Weight loss can also be observed as well as gastrointestinal obstruction, anemia, jaundice, and, less commonly, pancreatitis, which was also present in our patient who had elevation of her lipase [4]. The most typical locations for SPNs are the body and the tail of the pancreas [2]. Ultrasound, CT, and MRI may demonstrate a well-demarcated mass that can range from cystic to primarily solid. Imaging classically shows a thick peripheral capsule with or without calcifications [3, 4]. The above-mentioned imaging modalities can more accurately determine if there are findings that increase the risk of malignancy, including diameter greater than 3 cm, a solid component within the cyst, and main pancreatic duct dilation [4]. If the CT or MRI has findings indicative for increased risk of malignancy, as indicated above, endoscopic ultrasound with fine needle biopsy is recommended [4]. After fluid is obtained via biopsy it should be analyzed for cytology, tumor markers, and molecular markers. The composition of SPNs is typically a combination of cystic, necrotic, and hemorrhagic components [4]. Cytology for SPNs reveals characteristic branching papillae with myxoid stroma [4]. The molecular marker specific to SPNs is the CTNNB1 genetic mutation [2]. Unlike the more malignant and aggressive pancreatic cancers, such as adenocarcinomas seen mainly in older adults, SPNs have a favorable prognosis when complete resection is performed [4]. The outcomes are especially favorable in pediatric patients where the 5-year survival rate for those undergoing complete resection approaches 100% [3]. For this reason, resection is the definitive treatment, even in small children [3]. Timely diagnosis of this rare neoplasm is important because those patients that are able to have complete resection have a chance at excellent long-term survival.

## 4. Conclusion

Solid pseudopapillary neoplasms are a rare form of pancreatic cystic neoplasm that typically presents in young women and are very rare in children, with an incidence rate of 0.005–0.01 cases per 100,000 [3]. These lesions often present as an asymptomatic tumor found on incidental imaging. When symptomatic they typically present with abdominal pain, palpable abdominal mass, nausea, and vomiting [3]. Appropriate workup includes imaging with a MRCP which classically reveals the characteristic mixed solid and cystic lesions with or without calcifications and a thick peripheral capsule [3, 4]. Workup for this lesion includes confirmation of the diagnosis by endoscopic ultrasound and fine needle biopsy [4]. Timely diagnosis of this rare neoplasm may lead to resection and excellent long-term survival.

## Figures and Tables

**Table 1:** Clinical data, ranging from 53 to 183 cases, of solid pseudopapillary tumour of the pancreas from the pediatric literature [3].

Category	Findings
Age (years) [SD]	Mean: 13.1 range: 8–18

Sex	Male: 19.2%
	Female: 80.8%

Presentation	Abdominal pain: 48.1%
	Palpable mass: 25.1%
	Vomiting: 6.6%
	Trauma: 7.1%
	Dyspepsia: 3.8%
	Incidental: 2.7%
	Other: 6.6%

Location	Head, neck, and/or body: 45%
	Body and/or tail: 52.7%
	Unknown: 2.3%

Size, largest diameter (cm) [SD]	Mean: 9.3, range: 1–20

Operation	PPPD: 13%
	Whipple: 12.2%
	Duodenum-sparing head resection: 4.6%
	Distal pancreatectomy: 35.1%
	Local resection: 4.6%
	Enucleation: 2.3%
	Other: 4.6%
	Unspecified: 23.7%

Metastasis	3.1%

Follow-up (months) [SD]	Mean: 62.7, range: 6–240

Recurrence	8.7%

Time to recurrence (months) [SD]	Mean: 43.5, range: 0–96

Mortality	1
